# Immunonutrition: Feeding the gut, skin, and immune system

**DOI:** 10.5415/apallergy.0000000000000262

**Published:** 2026-02-16

**Authors:** Klaudia Ryczaj, Ruby Pawankar, Carina Venter

**Affiliations:** 1 Pediatric Teaching Hospital of the Medical University of Warsaw, Warsaw, Poland; 2 Nippon Medical School, Tokyo, Japan; 3 University of Colorado/Children’s Hospital Colorado, Aurora, CO

**Keywords:** Diet, epithelial barrier, gut-skin axis, immunonutrition, infants

## Abstract

**Background:**

Early life is a critical period for the maturation of the gut microbiota, epithelial barriers, and immune system. Disruption of gut and skin barrier integrity during this window is increasingly recognized as a key mechanism contributing to the development of allergic diseases.

**Objective:**

This review summarizes evidence on how infant dietary patterns and macronutrients influence epithelial barriers, microbiota development, and immune programming for allergy prevention.

**Methods:**

A comprehensive search and analysis of current literature regarding macronutrients, including carbohydrates and fiber, proteins, and fats, dietary diversity, and Western-type dietary patterns, was conducted to explore their collective effects on epithelial barrier function, microbiota, and immune outcomes during pregnancy and early life. Particular attention was given to complementary feeding.

**Results:**

Dietary composition significantly influences epithelial barrier integrity and immune development through direct nutritional effects and microbiota-mediated pathways. Dietary fibers and human milk oligosaccharides promote short-chain fatty acid production and microbial diversity, strengthening epithelial junctions, mucus secretion, and regulatory immune responses. Adequate protein intake supports epithelial renewal, while microbial metabolism of amino acids such as tryptophan and branched-chain fatty acids enhances barrier integrity and immune homeostasis. Dietary fats critically shape epithelial lipid composition, with balanced omega-3 and omega-6 fatty acids supporting barrier resilience, whereas excess saturated and trans fats promote dysbiosis and inflammation. Furthermore, greater dietary diversity during pregnancy and infancy correlates with enhanced microbial maturation and reduced atopic risk. Conversely, Western-type diets rich in ultra-processed foods and additives disrupt epithelial barriers and promote pro-inflammatory immune programming with long-lasting consequences beyond infancy.

**Conclusions:**

Optimizing maternal and early life nutrition through breastfeeding support, timely and diverse complementary feeding, adequate intake of fiber, high-quality proteins and fats, and avoidance of Western-type dietary patterns represents a promising strategy to support gut and skin barrier and to reduce the risk of allergic diseases across the lifespan.

## 1. Introduction

The global prevalence of allergic diseases has reached epidemic proportions over the last few decades, necessitating a deeper understanding of the mechanisms underlying immune dysregulation. Central to this understanding is the epithelial barrier theory [1], which proposes that the rise in allergic and autoimmune conditions is driven by the disruption of the specialized cell layers that protect the body from the external environment. These barriers, primarily in the gut and the skin, serve as the first line of defense and the primary site for immune education. When their integrity is compromised by environmental stressors, the resulting increase in permeability allows for the translocation of antigens and toxins, triggering chronic systemic inflammation and the progression of the atopic march.

The relationship between these protective surfaces is further elucidated by the dual allergen exposure hypothesis [2]. This concept posits that allergic sensitization occurs when the immune system encounters allergens through a compromised skin barrier, whereas early and controlled exposure through the gastrointestinal tract promotes the induction of oral tolerance. This highlights the importance of maintaining a healthy intestinal barrier to ensure that the immune system develops appropriate responses to dietary antigens. Consequently, the health of the gut and skin is linked through a complex communication network known as the gut-skin axis [3].

Recent advances in immunonutrition highlight how specific dietary components and dietary patterns can influence the immune response and support the physical structure of epithelial barriers [4]. This interaction is further characterized by the concept of trained immunity, where nutritional and microbial metabolites have long-lasting effects on the innate immune system through the functional reprogramming of myeloid cells [5]. These metabolic signals foster long-term resilience or, in the context of poor nutrition, prime the organism for chronic inflammatory conditions, establishing a baseline for systemic health or disease susceptibility from early life.

The first 1,000 days of life (pregnancy till 1 year of age) represent a critical window for the establishment of the gut microbiota and the maturation of the immune system. During this highly plastic period, the timing and quality of nutritional interventions are paramount and act as primary determinants of microbial succession and immune programming [6].

**Figure 1.**
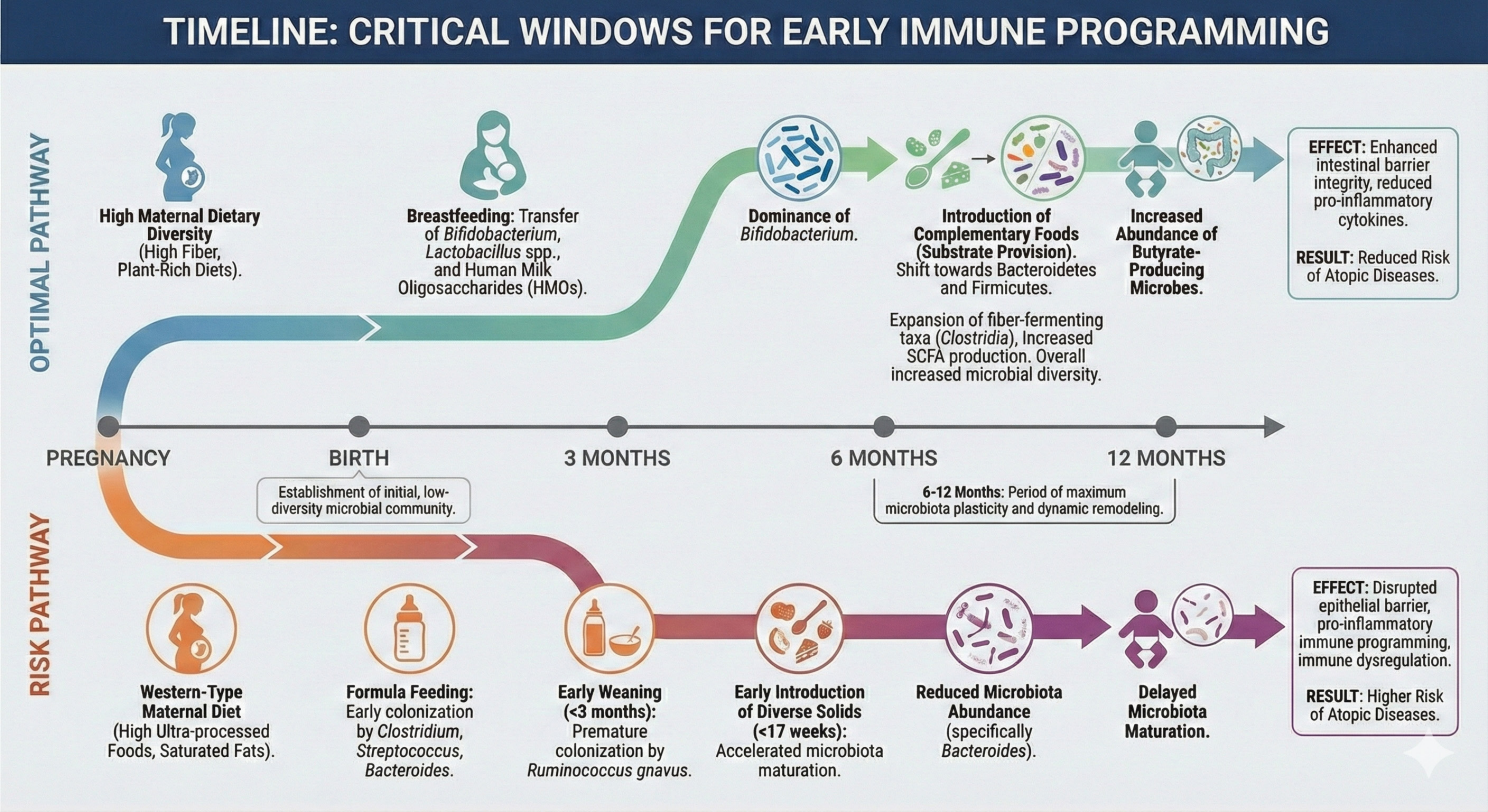
Timelines in allergy development. The figure demonstrates the timelines in allergy development over the life course. The infographics were generated with the assistance of Google Gemini AI based on scientific materials provided by the authors. All final outputs were critically reviewed and validated by the authors to ensure accuracy and fidelity to the source content.

This paper explores how immunonutrition strategies can be used to support these developmental processes, with a specific focus on the role of macronutrients, including proteins, fats, and carbohydrates during the first 1,000 days (Fig. 1). It further examines the importance of dietary diversity and the impact of Western-type dietary patterns. By exploring the link between nutrition, the microbiota, and the epithelial barriers, this work highlights the potential of early life nutrition to ensure long-term health.

## 2. Feeding the gut and the immune system

### 2.1. The intestinal epithelial barrier and microbiota

The human intestine represents the largest interface between the external environment and the host immune system. In infants and children, whose immune and epithelial systems are undergoing rapid maturation, the integrity of the intestinal epithelial barrier is crucial for establishing immune tolerance and protecting against inflammatory and allergic diseases. This barrier is composed of a single layer of epithelial cells covered by a protective mucus layer, the thickness and permeability of which critically determine host–microbe interactions and immune regulation. Structural integrity is maintained by intercellular tight junction (TJ) complexes, including claudin-1, occludin, and zonula occludens-1, which precisely regulate paracellular permeability [7, 8].

The functionality of this barrier is governed by the gut microbiota, a metabolically active ecosystem comprising more than 1,000 recognized species [9]. During early life, this microbial community plays a decisive role in host metabolism and the regulation of the maturing immune system [7, 10]. Diet plays a fundamental role in shaping the composition, diversity, and metabolic activity of the gut microbiota. Beyond the microbial community itself, microbiota-derived metabolites such as short-chain fatty acids (SCFAs), secondary bile acids, and tryptophan metabolites are key mediators of host–microbe mutualism. A balanced gut microbiota and its metabolic products contribute to vital physiological processes, including nutrient metabolism, the maintenance of epithelial architecture, systemic immune regulation, and protection against pathogenic colonization [11].

### 2.2. The immune system

The gut-associated lymphoid tissue (GALT) represents the largest and most complex immune organ in the human body and contains approximately 90% of all immunologically active cells. This extensive network functions as the principal site where the immune system is continuously educated by dietary antigens, commensal microorganisms, and microbiota-derived metabolites. Consequently, the GALT acts not only as a local defensive structure but also as a systemic regulator of immune homeostasis with important effects on inflammatory and allergic responses. Integrity of the intestinal epithelial barrier is fundamental for regulating immune responses within the gut. Any disruption of this architecture leads to increased intestinal permeability, which allows the translocation of dietary antigens and endotoxins such as lipopolysaccharides (LPS) into the systemic circulation. This process activates innate immune signaling pathways, including NF-κB, and induces the release of pro-inflammatory cytokines such as tumor necrosis factor alpha (TNF-α), interleukin (IL)-6, and IL-1β [7].

Loss of epithelial integrity further facilitates immune deviation toward type 2 inflammatory pathways by increasing antigen uptake and disrupting the induction of oral tolerance. This pathological process promotes the activation of group 2 innate lymphoid cells (ILC2), mast cells, basophils, and dendritic cells, ultimately favoring the Th2 polarized immune responses that underlie the development of allergic diseases [8]. Under homeostatic conditions, a healthy microbiota supports the differentiation of regulatory T cells (Tregs), which secrete anti-inflammatory cytokines like IL-10 and transforming growth factor beta to suppress such allergic deviations. However, gut microbiome dysbiosis can impair this balance by disrupting mucosal dendritic cell function, potentially through altered communication between group 3 innate lymphoid cells (ILC3) and dendritic cells [12, 13]

Beyond these local interactions and inflammatory pathways, recent advances in immunonutrition have highlighted the concept of trained immunity, where nutritional and microbial metabolites exert long-lasting effects on the innate immune system through the functional reprogramming of myeloid cells. While bacterial metabolites such as butyrate potentially program hematopoietic stem cells in the bone marrow to ensure a balanced immune response, Western-type dietary patterns induce systemic inflammation and innate immune reprogramming via the activation of the NLRP3 inflammasome, resulting in a persistent pro-inflammatory state that may remain even after subsequent dietary improvement. Such systemic modulation emphasizes that the nutritional environment during infancy acts as a primary determinant of long-term health, where beneficial metabolites foster resilience while Western-type dietary components may prime the organism for chronic inflammatory and allergic conditions across the entire lifespan [5, 14, 15].

### 2.3. Critical windows for early immune programming: breastfeeding and complementary foods

The first 1,000 days of life represent an important window for microbial colonization, maturation of the intestinal barrier, and the programming of the immune system [6]. Increasing evidence from both animal and human studies suggests that the developing gut microbiome plays a central role in the establishment of immune tolerance to food antigens [13, 16]. The development begins shortly after birth and reaches a stable state similar to that of adults by 3 to 5 years of age as it changes from a simple community into a complex and diverse ecosystem [6, 17, 18]. Microbial acquisition during infancy is influenced by a range of factors, including prenatal maternal microbial exposure, mode of delivery, maternal skin contact, and the gradual introduction of complementary foods. Early exposure to a diverse microbial ecosystem plays a fundamental role in shaping immune tolerance and competence, whereas disruptions of this process may impair immune maturation and increase the risk of inflammatory and allergic diseases later in life [6].

Breastfeeding and the composition of the complementary diet have emerged as one of the strongest determinants of gut microbiota development. Breastfeeding facilitates the transfer of beneficial bacteria, including *Bifidobacterium* and *Lactobacillus* species, while providing human milk oligosaccharides (HMOs) as essential bioactive components for microbial growth. These oligosaccharides serve as selective substrates for specific *Bifidobacterium* strains, which act as the primary producers of aromatic lactic acids within the infant gut. Among these metabolites, indole-3-lactic acid (ILA) is particularly meaningful for its ability to modulate cytokine responses in Th17 polarized cells. This interaction leads to an increased production of IL-22, which enhances the secretion of antimicrobial proteins and regulates both intestinal permeability and mucus production while simultaneously decreasing the levels of the pro-inflammatory cytokine IL-12p70 [19–22]. Recent evidence further suggests that the early life transmission of these *Bifidobacterium* species enhances the production of aromatic lactates, which actively suppress the production of IgE and are associated with a markedly reduced risk of food sensitization and atopic dermatitis during the first 5 years of life [23]. The American Academy of Pediatrics reports that exclusive breastfeeding for at least 3 months is associated with protection against early wheezing and a reduced risk of atopic dermatitis [24]. In contrast, formula-fed infants more frequently harbor *Clostridium*, *Streptococcus*, and *Bacteroides* species, which have been associated with increased rates of food sensitization [13].

Additionally, colostrum appears to play a particularly critical role in early immune and microbiota programming, delivering high concentrations of growth factors, immunoglobulins, antimicrobial peptides, and microbiota-shaping molecules. A recent study demonstrated that partial colostrum feeding was associated with a higher risk of peanut allergy and multiple food allergies compared with exclusive colostrum feeding [25]. Notably, no cases of peanut allergy were observed among infants receiving at least 9 colostrum feeds per day during the first 72 hours of life, and exclusive colostrum feeding remained protective regardless of the timing of peanut introduction [25].

Diet is a primary driver of microbiota maturation during the complementary feeding period as the introduction of solid foods provides new substrates for microbial growth and fermentation [26, 27]. This transition is characterized by a shift from *Bifidobacterium*-dominant communities toward increased representation of *Bacteroidetes* and *Firmicutes*. Importantly, this diversification enables the expansion of fiber-utilizing taxa such as *Clostridia* and results in increased production of immunomodulatory microbial metabolites, including SCFAs [12, 27–29]. Experimental evidence from murine models shows that early life microbiota composition, especially *Clostridia* and their metabolites, is essential for the induction of oral tolerance [30]. Certain bacterial taxa, including *Blautia* and *Phascolarctobacterium faecium*, have also been associated with protection against allergic sensitization, underscoring the importance of microbial diversity and functional capacity in maintaining immune health [30].

The period between 6 and 12 months of age represents the most dynamic phase of microbiota remodeling [27]. Alterations in the pace of microbial maturation have been linked to allergic outcomes, as both premature and delayed introduction of complementary foods may disrupt this finely tuned developmental trajectory. For instance, higher relative abundances of *Bifidobacterium* at 6 months of age are associated with a reduced risk of atopic dermatitis and lower rates of skin prick test positivity [31] Conversely, early introduction of solid foods before 3 months of age has been associated with premature colonization by taxa such as *Ruminococcus gnavus* and enrichment of metabolic pathways, which are linked to immune dysregulation and increased asthma risk [32]. Similarly, the introduction of highly diverse solid foods before 17 weeks of age or accelerated microbiota maturation between 3 and 6 months have been associated with an increased risk of allergic disease later in childhood [33, 34].

Delayed microbiota maturation at 12 months has also been identified as a shared feature across multiple pediatric allergic conditions [35, 36]. Reduced abundance of *Bacteroides* in early infancy correlates with subsequent asthma development, while food allergy has been linked to a reduced microbiota for age Z score during the first year of life [35, 37]. In contrast, an increased abundance of microbes that synthesize butyrate by the age of 1 year correlates with a lower incidence of atopic dermatitis and fewer positive results in skin prick tests [31].

Interestingly, the foundation for this development may begin even earlier, as acetate produced by the maternal microbiota during pregnancy can cross the placenta and influence fetal immune development [38, 39].

Collectively, these findings indicate that both the timing of complementary food introduction and the quality of microbial maturation are critical determinants of immune tolerance [40]. Together, maternal and infant microbiomes during the first 1,000 days of life play a central role in shaping immune trajectories and determining susceptibility to the atopic march, where an inability to develop immune tolerance connects early allergic sensitization with the subsequent emergence of atopic dermatitis and asthma [12, 27] (Fig. 1).

### 2.4. Macronutrients

#### 2.4.1. Carbohydrates and fiber

Diet-derived microbial metabolites play a central role in strengthening the intestinal epithelial barrier and shaping immune tolerance during early life. Among these metabolites, SCFAs, primarily acetate, propionate, and butyrate, are produced through the fermentation of dietary fibers and HMOs. These molecules exert pleiotropic effects on both epithelial and immune cells [41].

SCFAs regulate the expression of genes involved in barrier integrity and promote the production of tolerogenic cytokines. Experimental studies demonstrate that butyrate enhances the expression of TJ proteins and stimulates the production of mucus-associated proteins such as MUC2 and MUC5AC, as well as antimicrobial peptides including human β-defensin-3 [8, 28, 42–44]. These metabolites lower the intestinal luminal pH, creating an environment that restricts the growth of pH-sensitive pathogens, while simultaneously enhancing nutrient absorption [45]. Additionally, SCFAs exert immunoregulatory effects through activation of G protein-coupled receptors, inhibition of histone deacetylases, and modulation of Toll-like receptor (TLR) signaling. These pathways promote differentiation of Tregs and secretion of anti-inflammatory cytokines like IL-10, thereby inhibiting the production of pro-allergic cytokines such as IL-4, IL-5, and IL-13 [41, 44, 46].

Furthermore, butyrate can directly modulate type 2 immunity by restricting the differentiation of tuft cells and blocking the ILC2 pathway, effectively dampening the allergic inflammatory milieu. These metabolites also increase lymphocyte and leukocyte counts in GALT, and promote intestinal IgA secretion [12, 47]. Systematic reviews indicate that higher levels of SCFAs in the first few years of life have a protective effect against allergic diseases, especially for atopic dermatitis, wheeze, asthma, and IgE-mediated food allergy in childhood [42]. Conversely, reduced microbial diversity and lower levels of these metabolites disrupt these mechanisms and are associated with an increased risk of atopic diseases [12, 48].

Maternal diet during pregnancy also contributes to early immune programming. Microbial metabolites like acetate can cross the placenta and promote fetal Treg development, providing protection against postnatal allergic sensitization [12, 13]. Consistently, higher maternal fiber intake and diets rich in plant-based foods are associated with greater microbial diversity and reduced allergy risk in offspring [49]. Collectively, adequate dietary fiber intake and the resulting production of SCFAs represent a critical axis linking nutrition, microbiota, and immune tolerance. Disruption of this axis through dysbiosis or insufficient fiber intake impairs epithelial function and increases susceptibility to the atopic march.

#### 2.4.2. Proteins

Amino acids serve as the fundamental building blocks of proteins and are integral to the regulation of both adaptive and innate immune responses. These molecules govern the activation of B cells, T cells, natural killer cells, and macrophages, while remaining essential for lymphocyte proliferation and the synthesis of antibodies, cytokines, and cytotoxic molecules [50]. Research indicates that high-protein diets, regardless of whether the source is animal or plant-based, can reduce levels of pro-inflammatory adipokines such as chemerin and progranulin [51]. Conversely, protein deficiency leads to restricted amino acid availability, which induces cellular stress and activates T cells that release pro-inflammatory cytokines, thereby compromising immune homeostasis [52].

Adequate dietary protein intake is essential for intestinal epithelial renewal and innate immune defense, partly through the induction of antimicrobial peptide expression. Protein availability supports rapid epithelial turnover and contributes to the maintenance of barrier integrity during critical periods of growth and immune maturation [53].

Beyond its structural role, dietary protein significantly influences gut microbiota composition and serves as a substrate for microbial fermentation. In the gut, dietary protein undergoes metabolism by the microbiota, a process that has been associated with an increased abundance of *Bacteroides* species. This metabolic pathway results in the production of various bioactive compounds, including SCFAs, branched-chain fatty acids (BCAAs), and indoles. Specifically, BCAAs enhance gut barrier function by increasing mucus secretion and decreasing luminal pH, which collectively protect the intestinal lining from pathogenic encroachment. These metabolites, alongside SCFAs, contribute to improved gastrointestinal function and reduced systemic inflammation. Furthermore, they support metabolic health by improving insulin sensitivity and promoting fatty acid oxidation, thereby maintaining a healthy gut environment and supporting overall immune resilience [45]. Among specific amino acids, tryptophan plays a distinctive immunoregulatory role through its microbial metabolism. Commensal bacteria convert dietary tryptophan into a range of indole derivatives, including indole-3-propionic acid (IPA) and ILA. These metabolites enhance barrier function by increasing transepithelial electrical resistance, upregulating TJ proteins, and stimulating goblet cell activity to reinforce the mucus layer [54, 55]. Furthermore, indole derivatives suppress the epithelial expression of pro-inflammatory cytokines such as IL-8 and regulate the expression of the IL-10 receptor, which supports tissue repair and immune tolerance [12]. Specifically, IPA alleviates inflammatory injury in colonic epithelial cells by inhibiting apoptosis and blocking the release of pro-inflammatory cytokines such as IL-1β, IL-6, and TNF-α. These protective effects are mediated through the regulation of the TLR4 signaling pathways, including the NF-κB and TRIF cascades [55]. Tryptophan-derived metabolites also act as ligands for the aryl hydrocarbon receptor (AhR), which is expressed by epithelial cells and ILC3s. Activation of this receptor promotes cell survival and induces the production of IL-22, a cytokine critical for antimicrobial peptide production and barrier maintenance [12, 13]. Experimental and clinical evidence further suggests that AhR signaling supports the development of Tregs and suppresses driven mast cell activation. A deficiency of microbial tryptophan metabolites in early life is associated with impaired barrier integrity and increased susceptibility to severe allergic phenotypes, including multiple food allergies [12, 13].

Higher protein intake is associated with increased microbial diversity, particularly when derived from plant sources such as pea protein, which promotes the growth of beneficial taxa, including *Bifidobacterium* and *Lactobacillus*. These shifts in the microbial community reduce the presence of potentially pathogenic species like *Bacteroides fragilis* and *Clostridium perfringens* [56, 57]. This nutritional variety supports the coordinated maturation of the microbiota and the immune system during the most dynamic phases of early development.

#### 2.4.3. Fats

The quality of dietary fat plays a crucial role in maintaining intestinal barrier integrity and regulating immune responses.

Omega-3 polyunsaturated fatty acids (PUFA) support epithelial barrier function and exert anti-inflammatory effects by serving as precursors for specialized pro-resolving mediators that limit excessive immune activation [58]. These fatty acids modulate inflammation by competing with omega-6 fatty acids for enzymatic pathways, thereby reducing the synthesis of pro-inflammatory mediators such as prostaglandins and leukotrienes [59, 60]. Specifically, eicosapentaenoic acid (EPA) and docosahexaenoic acid (DHA) lower the activation of inflammatory transcription factors such as NF-κB and reduce the levels of pro-allergic cytokines including IL-4 and IL-13 [33, 61–63]. Furthermore, DHA suppresses responses, enhances activity, and supports the development of Tregs, which promotes the immune balance critical in preventing atopic dermatitis [64, 65]. Dietary fat composition also strongly influences the gut microbiota, as the intake of omega-3 fatty acids is associated with favorable microbial profiles, including an increased abundance of *Bifidobacterium*, *Lactobacillus*, and *Akkermansia muciniphila*, which are linked to improved mucosal integrity and metabolic homeostasis [56].

Regarding omega-6 fatty acids, their metabolites are vulnerable to oxidative stress from environmental factors, which can activate pro-inflammatory pathways involving eicosanoids such as thromboxane A2, prostaglandin E2, and leukotriene B4. These compounds contribute to stimulating the production of IgE and TNF, further amplifying inflammatory responses [66].

Monounsaturated fatty acids (MUFAs), primarily represented by oleic acid (OA) in olive oil and avocados, reduce the expression of NF-κB and decrease neutrophil elastase levels, thereby contributing to the mitigation of chronic inflammation and tissue degradation [67, 68].

In contrast, diets high in saturated fatty acids (SFAs), predominantly found in fatty meats, butter, and palm oil, have been shown to reduce the expression of TJ proteins and exacerbate epithelial barrier dysfunction [69]. Diets rich in saturated fats promote the expansion of pro-inflammatory bacteria, including *Bilophila* species, and disrupt the balance of key commensal communities [56]. High dietary intake of SFAs can activate TLR on immune cells, triggering the release of pro-inflammatory cytokines such as TNF-α, IL-6, and IL-1β. This process is also associated with increased production of reactive oxygen species (ROS) and impaired antioxidant defenses, contributing to tissue damage [70–72]. These fats have been linked to the activation of ILC3s and the subsequent production of IL-17 and IL-22 [70, 72]. Saturated fat consumption also induces the production of TSLP, IL-25, and IL-33, all of which promote responses characteristic of allergic diseases [73].

Similarly, trans fatty acids (TFAs) found in hydrogenated vegetable oils and processed foods are strongly implicated in pro-inflammatory effects and immune dysfunction. These fats contribute significantly to oxidative stress and activate TLR, leading to elevated plasma levels of inflammatory biomarkers such as IL-6 and E-selectin [74–76]. The consumption of industrial TFAs exacerbates microbial dysbiosis by increasing *Proteobacteria* and reducing beneficial groups such as *Bacteroidetes* and members of the *Lachnospiraceae* family [77].

Finally, maternal dietary fat intake during pregnancy may influence immune programming in the offspring. Higher maternal consumption of meat and saturated fats has been associated with a higher risk of atopic diseases in children, suggesting that early exposure to unfavorable lipid profiles contributes to long-term susceptibility to allergic disease [78, 79].

Collectively, these findings underscore that the balance of dietary fats is a critical determinant of mucosal and systemic immune health throughout the first 1,000 days of life.

### 2.5. Western-type diets

Western-type dietary patterns, characterized by high intake of fructose, saturated fats, and highly processed foods, have been consistently associated with impaired intestinal barrier function and adverse immune outcomes. Experimental studies demonstrate that such diets compromise goblet cell function and reduce mucus layer thickness, leading to increased intestinal permeability and enhanced exposure of the immune system to luminal antigens [80]. In pediatric nutrition, frequent consumption of ultra-processed foods (UPFs) and commercially processed infant products, often containing added acids or sweeteners, may further exacerbate barrier dysfunction during critical periods of immune development [81].

Food additives commonly present in Western-type diets, including emulsifiers such as carboxymethylcellulose and polysorbate 80, as well as artificial sweeteners, have been shown to disrupt the mucus microbiota interface. This disruption promotes bacterial encroachment toward the epithelium, induces low-grade inflammation, and interferes with the development of oral tolerance [82]. Animal studies provide additional evidence that maternal intake of dietary emulsifiers induces persistent alterations in the offspring gut microbiota, resulting in increased susceptibility to inflammatory and metabolic disorders later in life. These effects are mediated by pro-inflammatory microbial shifts, premature closure of goblet cell-associated antigen passages, and long-lasting impairment of mucosal immune regulation [83].

The excessive consumption of UPFs, which are typically rich in additives, emulsifiers, and added sugars, is associated with marked alterations in gut microbiota composition. These dietary patterns reduce beneficial commensal bacteria while favoring the expansion of pro-inflammatory taxa [7, 82]. Western-type diets and high fructose intake have been shown to profoundly alter microbial metabolic capacity in experimental models, while added sugars and sugar-sweetened beverages further destabilize microbial homeostasis [80, 84, 85]. Diets high in fat also promote metabolic endotoxemia through increased production and translocation of LPS, which disrupt epithelial integrity, thin the mucus layer, and sustain chronic low-grade inflammation [77]. Maternal dietary patterns may additionally influence allergy risk in the offspring. Higher consumption of processed foods during pregnancy has been associated with an increased incidence of food allergy in children, suggesting that prenatal exposure to Western-type dietary components can affect immune programming [37].

Advanced glycation end products (AGEs) represent another mechanistic link between Western-type diets and allergic disease. These compounds, abundant in highly processed and heat-treated foods, can bind to their receptors and trigger oxidative stress and inflammatory signaling pathways. In the gut, AGEs may exacerbate inflammatory processes by modulating IL-33 and TSLP levels and disrupt epithelial barrier integrity, alter the Th1 and Th2 immune balance, and modify allergen structure and immunogenicity [82, 86]. In parallel, they can influence gut microbiota composition independently of receptor-mediated signaling, collectively contributing to dysregulated immune responses and increased susceptibility to food allergy [87].

The Western diet is also a major contributor to systemic oxidative stress [88]. Diets high in AGEs and SFAs promote oxidative stress, and elevated ROS levels contribute to the disruption of the epithelial barrier [82, 88]. Furthermore, the low intake of fruits and vegetables in these diets significantly diminishes the availability and efficacy of dietary antioxidants, impairing the skin’s capacity to mitigate oxidative damage [89]. Regular and excessive intake of simple sugars promotes sustained elevations in blood glucose levels, which are closely linked to the induction of chronic inflammatory cascades across various tissues [90].

Finally, the use of commercially processed infant foods, often high in added sugars or refined starches, may disrupt the developmental trajectory of the microbiome by favoring the growth of pro-inflammatory taxa and inhibiting the expansion of beneficial species like *Bifidobacterium* and *Bacteroides* [45, 91]. Furthermore, maternal high-fat and sugar intake during pregnancy and lactation significantly alters the functional capacity of the infant gut microbiome, thereby shaping long-term immune health [92].

### 2.6. Diet diversity

Dietary diversity is strongly associated with increased microbial diversity in the human gut, a feature consistently linked to immune regulation, metabolic flexibility, and resilience to inflammatory disease [33]. Early life appears to be a particularly sensitive period during which exposure to a broad range of foods supports microbial maturation and the development of immune tolerance. The Enquiring About Tolerance study demonstrated that greater diversity in the introduction of food allergens during the first year of life was associated with higher overall microbial diversity and increased abundances of *Proteaceae* and *Proteobacteria*, supporting the concept that early dietary diversity contributes to microbiota development and immune education [93].

Maternal diet represents an additional layer of influence on offspring microbiota and immune outcomes. High maternal dietary diversity and consumption of proanthocyanidin-rich fruits during pregnancy have been linked to increased production of hippuric acid, a microbial metabolite associated with a significantly reduced risk of food allergy in offspring. Maternal nutrient intake has also been shown to influence offspring DNA methylation patterns, cord blood cytokine profiles, and early life gut microbiota composition, highlighting the intergenerational impact of dietary exposures on immune programming [33].

Dietary diversity appears to exert its strongest effects during the complementary feeding period, when solid foods are first introduced and the gut microbiota undergoes rapid expansion and functional diversification [26]. Early exposure to a wide range of plant-based foods, including legumes such as peanuts and soy, as well as tree nuts and sesame seeds, has been shown to shape gut microbiota composition by 12 months of age and to support the establishment of fiber-utilizing and metabolically active microbial communities [31].

Observational studies consistently report that greater dietary diversity during the first year of life is associated with a lower risk of food sensitization and allergic diseases, although these associations have not yet been confirmed in randomized controlled trials [37, 59, 60]. Similarly, recent evidence suggests that greater overall dietary diversity during pregnancy is linked to a reduced risk of food allergy in offspring, although systematic reviews indicate that the evidence connecting global dietary patterns during pregnancy to allergy outcomes remains limited [61, 62].

Mechanistically, dietary diversity is thought to promote oral tolerance by expanding the repertoire of microbial-derived metabolites and reinforcing epithelial barrier function, thereby maintaining the gut as a resilient interface against excessive systemic immune activation and the downstream progression of the atopic march. Consistent with this concept, higher maternal intake of vegetables and yogurt has been associated with greater gut microbiome diversity and elevated butyrate levels, which may influence fetal immune development through epigenetic mechanisms [63, 94]. Several studies have also reported that higher consumption of fruits and vegetables during pregnancy is associated with a reduced risk of atopic diseases, including atopic dermatitis, potentially through combined effects on maternal microbiota composition and antioxidant-mediated modulation of immune responses [49, 95–99].

## 3. Feeding the skin

### 3.1. Skin structure and seasonal changes

The skin barrier is a complex and dynamic system that constitutes the primary interface between the human body and the external environment. This multilayered defense system is composed of the epidermis, dermis, and subcutaneous tissue, with the epidermis playing a dominant role in barrier function. The stratum corneum, the outermost layer of the epidermis, is the principal determinant of permeability control and protection against mechanical, chemical, and microbial insults.

Its barrier properties rely on the highly ordered organization of corneocytes embedded within a lipid matrix rich in ceramides, cholesterol, and free fatty acids. This lipid barrier is composed of these three components in an approximate 1:1:1 ratio, where ceramides account for nearly half of the total lipid mass [100]. Among epidermal free fatty acids, linoleic acid (LA), which is an essential omega-6 PUFA, and OA, a nonessential omega-9 MUFA, are the most predominant [101]. LA serves as a critical structural component of omega-hydroxy ceramides, which are indispensable for the organization of lamellar lipid bilayers. The integrity of the epidermis is further supported by natural moisturizing factors derived from filaggrin degradation and the maintenance of an acidic surface pH [102].

Seasonal variations profoundly affect this composition, as levels of LA and long-chain SFAs typically decrease during colder months while OA levels increase. This biochemical shift disrupts the structural stability of the stratum corneum and impairs lipid homeostasis, leading to increased skin dryness and a compromised defense against external insults [103].

### 3.2. Structural and functional differences in infant skin

Infant skin differs markedly from adult skin due to its developmental immaturity. The stratum corneum in neonates and young infants is thinner and less compact with higher water content. Keratinocyte differentiation, desmosomal connections, and TJ organization are not fully developed. Furthermore, the lipid profile of infant skin is characterized by lower levels of ceramides and free fatty acids, notably deficient in essential omega-6 fatty acids like LA, which is a critical precursor for omega-hydroxy ceramides. This results in increased permeability, increased transepidermal water loss (TEWL), and a less effective barrier function, making infants more susceptible to environmental stressors and inflammatory conditions [102]. In addition, the surface area-to-body weight ratio in infants is substantially higher than in adults, making infants particularly vulnerable to the percutaneous absorption of exogenous substances [104].

### 3.3. The gut-skin axis

The gut-skin axis represents a bidirectional communication network where the health of the gastrointestinal tract profoundly dictates the physiological state of the skin. When the intestinal barrier is compromised due to poor nutrition or dysbiosis, intestinal bacteria and their metabolites enter the bloodstream and accumulate in the skin, disrupting cutaneous homeostasis. This accumulation triggers the release of pro-inflammatory cytokines that can manifest as skin conditions such as eczema or increased sensitivity [105].

Conversely, a healthy gut microbiome produces beneficial metabolites like SCFAs that not only acidify the skin’s pH but also activate peroxisome proliferator-activated receptor-alpha. This activation enhances keratinocyte differentiation and lipid metabolism, reinforcing the physical resilience of the skin [3].

Within this axis, microorganisms influence skin health through their ability to metabolize dietary lipids. Bacteria such as *Lactobacillus plantarum* can transform PUFAs into hydroxy, oxo, conjugated, and partially SFAs, which enter the host circulation and modify systemic lipid profiles. These microbially derived lipids enter the host circulation and modify systemic lipid profiles, subsequently affecting epidermal lipid composition and inflammatory signaling [106].

### 3.4. Macronutrients

#### 3.4.1. Carbohydrates and fiber

Dietary carbohydrates, particularly nondigestible fermentable fibers, influence skin barrier integrity indirectly through modulation of the gut microbiota. SCFAs exert systemic anti-inflammatory effects and influence epidermal biology by promoting keratinocyte differentiation, enhancing mitochondrial metabolism, and supporting the synthesis of structural proteins and ceramides [107–109]. Furthermore, these microbial products play a role in shaping skin microbiome profiles and contribute to the acidification of the skin pH. This process occurs by mitigating inflammatory responses and decreasing the levels of TSLP [41, 108, 109]. Experimental models demonstrate that fiber-rich diets improve skin barrier function and attenuate allergic sensitization [110].

#### 3.4.2. Proteins

Proteins are the essential building blocks for structural proteins and natural moisturizing factors. The cornified cell envelope is a robust protein and lipid polymer structure composed of loricrin, involucrin, and small proline-rich proteins.

Beyond their structural role, certain amino acids exert immunomodulatory effects through microbial metabolism. Tryptophan, in particular, is converted by gut bacteria into indole derivatives that activate the AhR. Activation of this pathway promotes keratinocyte differentiation, supports immune homeostasis, and enhances skin barrier repair.

BCAAs contribute to epithelial resilience by modulating keratin expression. This regulation protects epithelial cells against mechanical and chemical stress, reinforcing the physical integrity of the tissue [111, 112].

#### 3.4.3. Fats

Dietary fats play a central role in shaping the lipid composition and functional integrity of the skin barrier. Alterations in lipid homeostasis, arising from inadequate intake or imbalance of dietary fats, may weaken epidermal barrier function, leading to increased TEWL and heightened vulnerability to inflammatory processes [108, 113].

In an optimal skin barrier, the omega-6 to omega-3 ratio is approximately 3:1. However, because these fats rely on the same enzymes, a high prevalence of omega-6 in modern diets can lead to an underrepresentation of omega-3 derivatives in skin lipid profiles [113].

LA, an essential omega-6 fatty acid, plays a vital structural role in the epidermis as a primary precursor for ceramides, which are crucial for building and maintaining the stratum corneum barrier [113]. Furthermore, gamma linolenic acid stands out for its anti-inflammatory properties as it enhances ceramide synthesis and promotes epidermal cell proliferation, thereby supporting effective barrier repair [114].

Omega-3 HMOs, including alpha linolenic acid, EPA, and DHA, contribute to epidermal lipid homeostasis by modulating inflammation and enhancing ceramide synthesis [115]. These lipids offer extensive benefits by modulating oxidative stress and protecting the skin from UV-induced damage [116]. Diets enriched in omega-3 fatty acids promote keratinocyte differentiation and have been shown to increase overall ceramide levels while enhancing filaggrin expression [113, 117].

Both omega-6 and omega-3 fatty acids are essential for improving hydration, reducing TEWL, and enhancing wound healing processes [75, 78, 116, 118, 119].

MUFAs also demonstrate significant antioxidant properties, contributing to their potential role in reducing oxidative stress and supporting skin health. These fats contribute to wound healing by promoting reepithelialization, enhancing collagen deposition, and supporting angiogenesis, all of which are essential for effective tissue repair [67, 68].

Finally, interactions between genetics and nutrition affect fatty acid metabolism. Variants in the FADS gene cluster influence long-chain HMO levels and eczema risk. For example, gestational fish intake affects DNA methylation and the expression of FADS1, FADS2, and ELOVL5 genes, linking maternal consumption to reduced allergy risk in offspring [120]. This highlights the importance of personalized nutritional strategies that account for both dietary intake and genetic predisposition.

### 3.5. Western-type diets

The Western-type diet serves as a primary contributor to systemic oxidative stress [88]. Diets rich in AGEs and SFAs promote oxidative stress through the elevation of mitochondrial reactive oxygen species, which directly leads to the disruption of the skin barrier [82, 88]. Furthermore, high-fat diets reduce the synthesis of ceramides and other essential lipids, a metabolic shift that is closely associated with dysbiosis in the cutaneous microbiome. Consequently, high-fat dietary patterns may impair the inherent healing capacity of the skin and exacerbate inflammatory responses by compromising the physical and biological integrity of the epidermal surface [77, 121, 122].

## 4. Conclusions

Early life represents a uniquely sensitive period during which nutrition, epithelial barrier maturation, microbiota development, and immune programming are tightly interconnected. The evidence synthesized in this review underscores that the gut and skin are not merely passive interfaces but dynamic immunological organs whose integrity is essential for the establishment of immune tolerance and long-term health.

**Figure 2.**
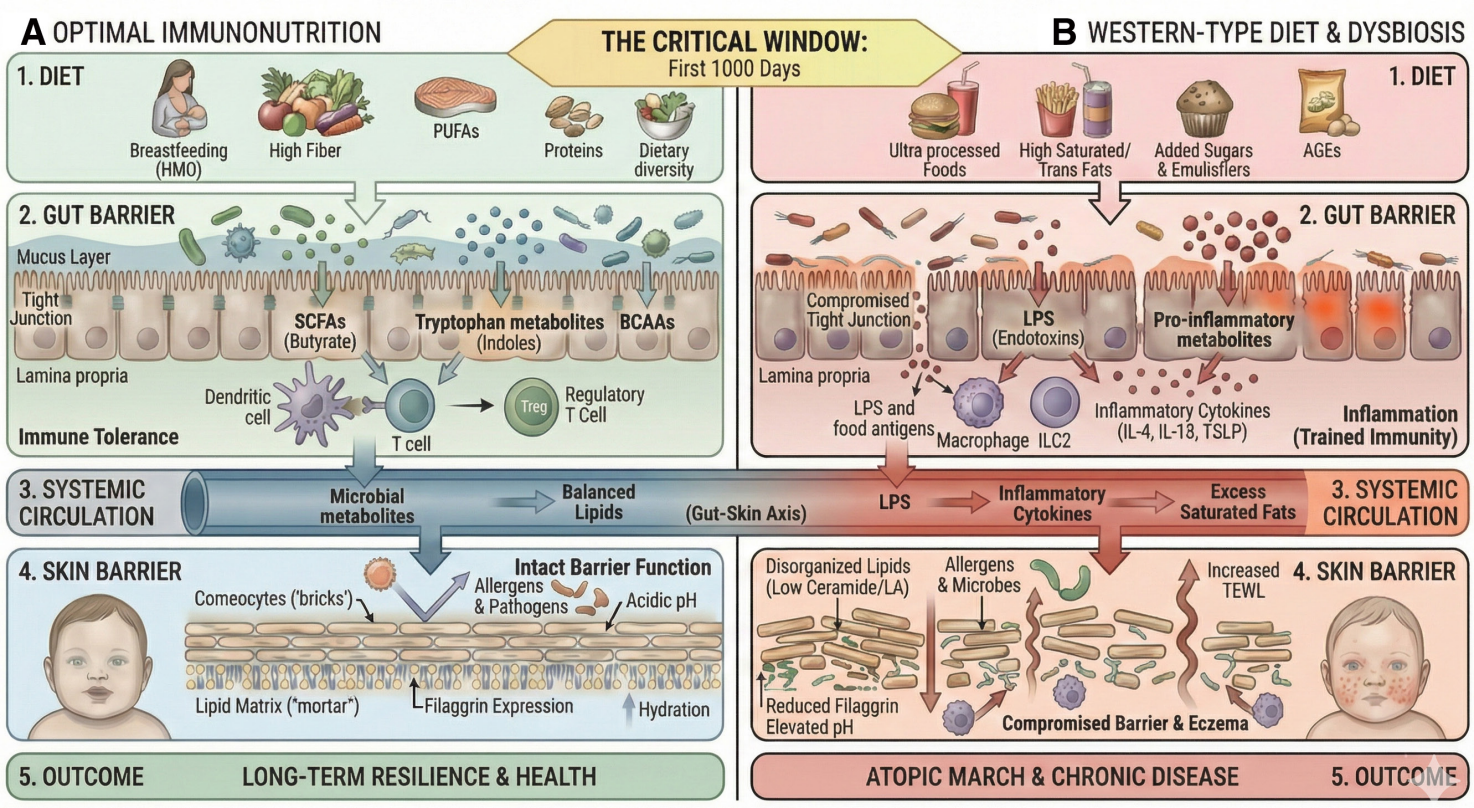
Dietary factors and allergy development. The figure demonstrates the role of a range of dietary factors in the development of allergic diseases. The infographics were generated with the assistance of Google Gemini AI based on scientific materials provided by the authors. All final outputs were critically reviewed and validated by the authors to ensure accuracy and fidelity to the source content.

Diet emerges as an important regulator of these processes. Macronutrients shape epithelial structure and function directly, while also acting indirectly through modulation of the gut microbiota and its metabolic output. Dietary fibers and HMOs support microbial diversity and the production of SCFAs, which strengthen epithelial junctions, promote mucus production, and foster regulatory immune pathways. Adequate protein intake supports epithelial renewal and immune competence, while microbial metabolism of amino acids such as tryptophan and the generation of BCAAs reinforce barrier integrity and immune homeostasis. Dietary fats play a particularly pivotal role by determining lipid composition of epithelial membranes and signaling pathways, with balanced intakes of omega-3 and omega-6 fatty acids supporting barrier resilience, while excess saturated and trans fats promote inflammation, dysbiosis, and epithelial dysfunction (Fig. 2).

The quality and diversity of the diet further modulate these effects. Greater dietary diversity during infancy and pregnancy consistently correlates with enhanced microbial maturation, expanded metabolic capacity, and reduced risk of atopic diseases.

In contrast, Western-type dietary patterns characterized by high intakes of UPFs, added sugars, saturated fats, and food additives disrupt epithelial barriers, impair microbial ecology, and promote pro-inflammatory immune programming. These effects may be amplified during critical windows of development, with long-lasting consequences that extend beyond infancy.

Collectively, the findings reviewed here underscore that feeding the epithelial barrier is, in essence, feeding the future of the human immune system.

## Conflicts of interest

The authors have no financial conflicts of interest.

## Author contributions

Klaudia Ryczaj wrote the first draft of the paper. Carina Venter and Ruby Pawankar edited and finalized the initial proof.

## References

[1] Pawankar R Canonica GW Holgate ST Lockey RF. Allergic diseases and asthma: a major global health concern. Curr Opin Allergy Clin Immunol. 2012;12:39-41. 22157151 10.1097/ACI.0b013e32834ec13b

[2] Lack G. Epidemiologic risks for food allergy. J Allergy Clin Immunol. 2008;121:1331-1336. 18539191 10.1016/j.jaci.2008.04.032

[3] Rios-Carlos M Cervantes-García D Córdova-Dávalos LE Bermúdez-Humarán LG Salinas E. Unraveling the gut-skin axis in atopic dermatitis: exploiting insights for therapeutic strategies. Gut Microbes. 2024;16:2430420. 39601281 10.1080/19490976.2024.2430420 PMC11610564

[4] Venter C. Immunonutrition: diet diversity, gut microbiome and prevention of allergic diseases. Allergy Asthma Immunol Res. 2023;15:545-561. 37827976 10.4168/aair.2023.15.5.545 PMC10570780

[5] Netea MG Domínguez-Andrés J Barreiro LB Chavakis T Divangahi M Fuchs E Joosten LAB van der Meer JWM Mhlanga MM Mulder WJM Riksen NP Schlitzer A Schultze JL Stabell Benn C Sun JC Xavier RJ Latz E. Defining trained immunity and its role in health and disease. Nat Rev Immunol. 2020;20:375-388. 32132681 10.1038/s41577-020-0285-6 PMC7186935

[6] Notarbartolo V Carta M Accomando S Giuffrè M. The first 1,000 days of life: how changes in the microbiota can influence food allergy onset in children. Nutrients. 2023;15:4014. 37764797 10.3390/nu15184014 PMC10534753

[7] Akdis CA. Does the epithelial barrier hypothesis explain the increase in allergy, autoimmunity, and other chronic conditions? Nat Rev Immunol. 2021;21:739-751. 33846604 10.1038/s41577-021-00538-7

[8] Paparo L Nocerino R Ciaglia E Di Scala C De Caro C Russo R Trinchese G Aitoro R Amoroso A Bruno C Di Costanzo M Passariello A Messina F Agangi A Napolitano M Voto L Gatta GD Pisapia L Montella F Mollica MP Calignano A Puca A Berni Canani R. Butyrate as a bioactive human milk protective component against food allergy. Allergy. 2021;76:1398-1415. 33043467 10.1111/all.14625 PMC8247419

[9] Adak A Khan MR. An insight into gut microbiota and its functionalities. Cell Mol Life Sci. 2019;76:473-493. 30317530 10.1007/s00018-018-2943-4 PMC11105460

[10] Forde B Yao L Shaha R Murphy S Lunjani N O’Mahony L. Immunomodulation by foods and microbes: unravelling the molecular tango. Allergy. 2022;77:3513-3526. 35892227 10.1111/all.15455 PMC10087875

[11] Wang J Zhu N Su X Gao Y Yang R. Gut-microbiota-derived metabolites maintain gut and systemic immune homeostasis. Cells. 2023;12:793. 36899929 10.3390/cells12050793 PMC10000530

[12] Davis EC Monaco CL Insel R Järvinen KM. Gut microbiome in the first 1,000 days and risk for childhood food allergy. Ann Allergy Asthma Immunol. 2024;133:252-261. 38494114 10.1016/j.anai.2024.03.010 PMC11344696

[13] Robbins E Koueik J Singh AM Frischmeyer-Guerrerio PA Hourigan SK. Role of the early-life microbiome in the development of food allergy. J Allergy Clin Immunol Pract. Published online 2025. doi: 10.1016/j.jaip.2025.12.007. 10.1016/j.jaip.2025.12.007 PMC12931096 41419086

[14] Fanucchi S Domínguez-Andrés J Joosten LAB Netea MG Mhlanga MM. The intersection of epigenetics and metabolism in trained immunity. Immunity. 2021;54:32-43. 33220235 10.1016/j.immuni.2020.10.011

[15] Christ A Günther P Lauterbach MAR Duewell P Biswas D Pelka K Scholz CJ Oosting M Haendler K Baßler K Klee K Schulte-Schrepping J Ulas T Moorlag SJCFM Kumar V Park MH Joosten LAB Groh LA Riksen NP Espevik T Schlitzer A Li Y Fitzgerald ML Netea MG Schultze JL Latz E. Western diet triggers NLRP3-dependent innate immune reprogramming. Cell. 2018;172:162-175.e14. 29328911 10.1016/j.cell.2017.12.013 PMC6324559

[16] Fujimura KE Sitarik AR Havstad S Lin DL Levan S Fadrosh D Panzer AR LaMere B Rackaityte E Lukacs NW Wegienka G Boushey HA Ownby DR Zoratti EM Levin AM Johnson CC Lynch SV. Neonatal gut microbiota associates with childhood multisensitized atopy and T cell differentiation. Nat Med. 2016;22:1187-1191. 27618652 10.1038/nm.4176 PMC5053876

[17] Donald K Finlay BB. Early-life interactions between the microbiota and immune system: impact on immune system development and atopic disease. Nat Rev Immunol. 2023;23:735-748. 37138015 10.1038/s41577-023-00874-w

[18] Stewart CJ Ajami NJ O’Brien JL Hutchinson DS Smith DP Wong MC Ross MC Lloyd RE Doddapaneni HV Metcalf GA Muzny D Gibbs RA Vatanen T Huttenhower C Xavier RJ Rewers M Hagopian W Toppari J Ziegler A-G She J-X Akolkar B Lernmark A Hyoty H Vehik K Krischer JP Petrosino JF. Temporal development of the gut microbiome in early childhood from the TEDDY study. Nature. 2018;562:583-588. 30356187 10.1038/s41586-018-0617-x PMC6415775

[19] Keir M Yi T Lu T Ghilardi N. The role of IL-22 in intestinal health and disease. J Exp Med. 2020;217:e20192195. 32997932 10.1084/jem.20192195 PMC7062536

[20] Henrick BM Rodriguez L Lakshmikanth T Pou C Henckel E Arzoomand A Olin A Wang J Mikes J Tan Z Chen Y Ehrlich AM Bernhardsson AK Mugabo CH Ambrosiani Y Gustafsson A Chew S Brown HK Prambs J Bohlin K Mitchell RD Underwood MA Smilowitz JT German JB Frese SA Brodin P. Bifidobacteria-mediated immune system imprinting early in life. Cell. 2021;184:3884-3898.e11. 34143954 10.1016/j.cell.2021.05.030

[21] Shibata R Nakanishi Y Suda W Nakano T Sato N Inaba Y Kawasaki Y Hattori M Shimojo N Ohno H. Neonatal gut microbiota and risk of developing food sensitization and allergy. J Allergy Clin Immunol. 2025;155:932-946. 39692676 10.1016/j.jaci.2024.10.029

[22] Seppo AE Bu K Jumabaeva M Thakar J Choudhury RA Yonemitsu C Bode L Martina CA Allen M Tamburini S Piras E Wallach DS Looney RJ Clemente JC Järvinen KM. Infant gut microbiome is enriched with *Bifidobacterium longum* ssp. infantis in Old Order Mennonites with traditional farming lifestyle. Allergy. 2021;76:3489-3503. 33905556 10.1111/all.14877 PMC9230048

[23] Myers PN Dehli RK Mie A Moll JM Roager HM Eriksen C Laursen MF Staudinger EM Chatzigiannidou I Johansen PLærke van Best N O’Hely M Andersen D Nørregaard NL Pedersen M Hamelmann E Lau S Bahl MI Abou Hachem M Licht TR Nielsen HB Thysen AH Vuillermin P Penders J Kristiansen K Scheynius A Alm J Brix S. Early-life colonization by aromatic-lactate-producing Bifidobacteria lowers the risk of allergic sensitization. Nat Microbiol. Published online 2026. 10.1038/s41564-025-02244-9 41526643

[24] Greer FR Sicherer SH Burks AW; COMMITTEE ON NUTRITION. The effects of early nutritional interventions on the development of atopic disease in infants and children: the role of maternal dietary restriction, breastfeeding, hydrolyzed formulas, and timing of introduction of allergenic complementary foods. Pediatrics. 2019;143:e20190281. 30886111 10.1542/peds.2019-0281

[25] Bhasin M Cooper M Macchiaverni P Joys RS O’Sullivan TA Keelan JA Venter C Palmer DJ Lowe AJ Prescott SL Silva D Verhasselt V. Colostrum as a protective factor against peanut allergy: evidence from a birth cohort. Allergy. Published online 2025:1-11. doi: 10.1111/all.70043. 40968490 10.1111/all.70043 PMC12862506

[26] Homann C-M Rossel CAJ Dizzell S Bervoets L Simioni J Li J Gunn E Surette MG de Souza RJ Mommers M Hutton EK Morrison KM Penders J van Best N Stearns JC. Infants’ first solid foods: impact on gut microbiota development in two intercontinental cohorts. Nutrients. 2021;13:2639. 34444798 10.3390/nu13082639 PMC8400337

[27] Ding M Ross RP Dempsey E Li B Stanton C. Infant gut microbiome reprogramming following introduction of solid foods (weaning). Gut Microbes. 2025;17:2571428. 41208251 10.1080/19490976.2025.2571428 PMC12604632

[28] Kim S Ndwandwe C Devotta H Kareem L Yao L O’Mahony L. Role of the microbiome in regulation of the immune system. Allergol Int. 2025;74:187-196. 39955207 10.1016/j.alit.2024.12.006

[29] Łoniewska B Fraszczyk-Tousty M Tousty P Skonieczna-Żydecka K Maciejewska-Markiewicz D Łoniewski I. Analysis of fecal short-chain fatty acids (SCFAs) in healthy children during the first two years of life: an observational prospective cohort study. Nutrients. 2023;15:367. 36678236 10.3390/nu15020367 PMC9864378

[30] Light SH Nagler CR. Regulation of immune responses to food by commensal microbes. Immunol Rev. 2024;326:203-218. 39285525 10.1111/imr.13396 PMC11472335

[31] Korpela K Hurley S Ford SA Franklin R Byrne S Lunjani N Forde B Neogi U Venter C Walter J Hourihane J O’Mahony L; CORAL Study Group. Association between gut microbiota development and allergy in infants born during pandemic-related social distancing restrictions. Allergy. 2024;79:1938-1951. 38419554 10.1111/all.16069

[32] Shenhav L Fehr K Reyna ME Petersen C Dai DLY Dai R Breton V Rossi L Smieja M Simons E Silverman MA Levy M Bode L Field CJ Marshall JS Moraes TJ Mandhane PJ Turvey SE Subbarao P Surette MG Azad MB. Microbial colonization programs are structured by breastfeeding and guide healthy respiratory development. Cell. 2024;187:5431-5452.e20. 39303691 10.1016/j.cell.2024.07.022 PMC11531244

[33] Venter C. Maternal diet and complementary food diversity on allergy prevention. BMJ Nutr Prev Health. 2023;6:s20-s30.

[34] Sausenthaler S Heinrich J Koletzko S; GINIplus and LISAplus Study Groups. Early diet and the risk of allergy: what can we learn from the prospective birth cohort studies GINIplus and LISAplus? Am J Clin Nutr. 2011;94:2012S-2017S. 21543544 10.3945/ajcn.110.001180

[35] Pawankar R Cezmi A. Climate change and the epithelial barrier theory in allergic diseases: a one health approach to a green environment. Allergy. 2023;78:2829-2834. 37675628 10.1111/all.15885

[36] Hoskinson C Dai DLY Del Bel KL Becker AB Moraes TJ Mandhane PJ Finlay BB Simons E Kozyrskyj AL Azad MB Subbarao P Petersen C Turvey SE. Delayed gut microbiota maturation in the first year of life is a hallmark of pediatric allergic disease. Nat Commun. 2023;14:4785. 37644001 10.1038/s41467-023-40336-4 PMC10465508

[37] Brandwein M Enten Vissoker R Jackson H Rogan T Pitcock J Krinkin E Venter C. Redefining the role of nutrition in infant food allergy prevention: a narrative review. Nutrients. 2024;16:838. 38542749 10.3390/nu16060838 PMC10974873

[38] Thorburn AN McKenzie CI Shen S Stanley D Macia L Mason LJ Roberts LK Wong CHY Shim R Robert R Chevalier N Tan JK Mariño E Moore RJ Wong L McConville MJ Tull DL Wood LG Murphy VE Mattes J Gibson PG Mackay CR. Evidence that asthma is a developmental origin disease influenced by maternal diet and bacterial metabolites. Nat Commun. 2015;6:7320. 26102221 10.1038/ncomms8320

[39] Hu M Eviston D Hsu P Mariño E Chidgey A Santner-Nanan B Wong K Richards JL Yap YA Collier F Quinton A Joung S Peek M Benzie R Macia L Wilson D Ponsonby A-L Tang MLK O’Hely M Daly NL Mackay CR Dahlstrom JE Vuillermin P Nanan R; BIS Investigator Group. Decreased maternal serum acetate and impaired fetal thymic and regulatory T cell development in preeclampsia. Nat Commun. 2019;10:3031. 31292453 10.1038/s41467-019-10703-1 PMC6620275

[40] Özçam M Lin DL Gupta CL Li A Gomez JC Wheatley LM Baloh CH Sanda S Jones SM Lynch SV. Gut microbial bile and amino acid metabolism associate with peanut oral immunotherapy failure. Nat Commun. 2025;16:6330. 40634275 10.1038/s41467-025-61161-x PMC12241578

[41] Venter C Meyer RW Greenhawt M Pali-Schöll I Nwaru B Roduit C Untersmayr E Adel-Patient K Agache I Agostoni C Akdis CA Feeney M Hoffmann-Sommergruber K Lunjani N Grimshaw K Reese I Smith PK Sokolowska M Vassilopoulou E Vlieg-Boerstra B Amara S Walter J O’Mahony L. Role of dietary fiber in promoting immune health—an EAACI position paper. Allergy. 2022;77:3185-3198. 35801383 10.1111/all.15430

[42] Sasaki M Suaini NHA Afghani J Heye KN O’Mahony L Venter C Lauener R Frei R Roduit C. Systematic review of the association between short-chain fatty acids and allergic diseases. Allergy. 2024;79:1789-1811. 38391245 10.1111/all.16065

[43] Nshanian M Gruber JJ Geller BS Chleilat F Lancaster SM White SM Alexandrova L Camarillo JM Kelleher NL Zhao Y Snyder MP. Short-chain fatty acid metabolites propionate and butyrate are unique epigenetic regulatory elements linking diet, metabolism and gene expression. Nat Metab. 2025;7:196-211. 39789354 10.1038/s42255-024-01191-9 PMC11774759

[44] Losol P Wolska M Wypych TP Yao L O’Mahony L Sokolowska M. A cross talk between microbial metabolites and host immunity: its relevance for allergic diseases. Clin Transl Allergy. 2024;14:e12339. 38342758 10.1002/clt2.12339 PMC10859320

[45] Ross FC Patangia D Grimaud G Lavelle A Dempsey EM Ross RP Stanton C. The interplay between diet and the gut microbiome: implications for health and disease. Nat Rev Microbiol. 2024;22:671-686. 39009882 10.1038/s41579-024-01068-4

[46] Roduit C Frei R Ferstl R Loeliger S Westermann P Rhyner C Schiavi E Barcik W Rodriguez-Perez N Wawrzyniak M Chassard C Lacroix C Schmausser-Hechfellner E Depner M von Mutius E Braun-Fahrländer C Karvonen AM Kirjavainen PV Pekkanen J Dalphin J-C Riedler J Akdis C Lauener R O’Mahony L; PASTURE/EFRAIM study group. High levels of butyrate and propionate in early life are associated with protection against atopy. Allergy. 2019;74:799-809. 30390309 10.1111/all.13660

[47] Ashkanani A Ashkanani G Yousef M Rob M Al-Marri M Naseem N Laws S’ad Chaari A. Microbiome and skin health: a systematic review of nutraceutical interventions, disease severity, inflammation, and gut microbiota. Microorganisms. 2026;14:63. 10.3390/microorganisms14010063 PMC12844480 41597583

[48] Hoskinson C Petersen C Turvey SE. How the early life microbiome shapes immune programming in childhood asthma and allergies. Mucosal Immunol. 2025;18:26-35. 39675725 10.1016/j.mucimm.2024.12.005

[49] Venter C Palumbo MP Glueck DH Sauder KA O’Mahony L Fleischer DM Ben-Abdallah M Ringham BM Dabelea D. The maternal diet index in pregnancy is associated with offspring allergic diseases: the healthy start study. Allergy. 2022;77:162-172. 34018205 10.1111/all.14949 PMC9292464

[50] Li P Yin Y-L Li D Kim SW Wu G. Amino acids and immune function. Br J Nutr. 2007;98:237-252. 17403271 10.1017/S000711450769936X

[51] Markova M Koelman L Hornemann S Pivovarova O Sucher S Machann J Rudovich N Thomann R Schneeweiss R Rohn S Pfeiffer AFH Aleksandrova K. Effects of plant and animal high protein diets on immune-inflammatory biomarkers: a 6-week intervention trial. Clin Nutr. 2020;39:862-869. 30967307 10.1016/j.clnu.2019.03.019

[52] Rubio-Patiño C Bossowski JP De Donatis GM Mondragón L Villa E Aira LE Chiche J Mhaidly R Lebeaupin C Marchetti S Voutetakis K Chatziioannou A Castelli FA Lamourette P Chu-Van E Fenaille F Avril T Passeron T Patterson JB Verhoeyen E Bailly-Maitre B Chevet E Ricci J-E. Low-protein diet induces IRE1α-dependent anticancer immunosurveillance. Cell Metab. 2018;27:828-842.e7. 29551590 10.1016/j.cmet.2018.02.009

[53] Tang Y Li J Liao S Qi M Kong X Tan B Yin Y Wang J. The effect of dietary protein intake on immune status in pigs of different genotypes. Food Agric Immunol. 2018;29:776-784.

[54] Li J Zhang L Wu T Li Y Zhou X Ruan Z. Indole-3-propionic acid improved the intestinal barrier by enhancing epithelial barrier and mucus barrier. J Agric Food Chem. 2021;69:1487-1495. 33356219 10.1021/acs.jafc.0c05205

[55] Chen Y Li Y Li X Fang Q Li F Chen S Chen W. Indole‑3‑propionic acid alleviates intestinal epithelial cell injury via regulation of the TLR4/NF‑κB pathway to improve intestinal barrier function. Mol Med Rep. 2024;30:189. 39219265 10.3892/mmr.2024.13313 PMC11350629

[56] Tomova A Bukovsky I Rembert E Yonas W Alwarith J Barnard ND Kahleova H. The effects of vegetarian and vegan diets on gut microbiota. Front Nutr. 2019;6:47. 31058160 10.3389/fnut.2019.00047 PMC6478664

[57] Laursen MF Andersen LBB Michaelsen KF Mølgaard C Trolle E Bahl MI Licht TR. Infant gut microbiota development is driven by transition to family foods independent of maternal obesity. mSphere. 2016;1:e00069-e00015. 10.1128/mSphere.00069-15 PMC4863607 27303699

[58] Seethaler B Lehnert K Yahiaoui-Doktor M Basrai M Vetter W Kiechle M Bischoff SC. Omega-3 polyunsaturated fatty acids improve intestinal barrier integrity-albeit to a lesser degree than short-chain fatty acids: an exploratory analysis of the randomized controlled LIBRE trial. Eur J Nutr. 2023;62:2779-2791. 37318580 10.1007/s00394-023-03172-2 PMC10468946

[59] Roduit C Frei R Loss G Büchele G Weber J Depner M Loeliger S Dalphin M-L Roponen M Hyvärinen A Riedler J Dalphin J-C Pekkanen J von Mutius E Braun-Fahrländer C Lauener R; Protection Against Allergy–Study in Rural Environments study group. Development of atopic dermatitis according to age of onset and association with early-life exposures. J Allergy Clin Immunol. 2012;130:130-136. 22521248 10.1016/j.jaci.2012.02.043

[60] Zhong C Guo J Tan T Wang H Lin L Gao D Li Q Sun G Xiong G Yang X Hao L Yang H Yang N. Increased food diversity in the first year of life is inversely associated with allergic outcomes in the second year. Pediatr Allergy Immunol. 2022;33:e13707. 34843132 10.1111/pai.13707

[61] Bodén S Lindam A Domellöf M Venter C West CE. Diet diversity in pregnancy and early allergic manifestations in the offspring. Clin Exp Allergy. 2023;53:963-968. 37271985 10.1111/cea.14346

[62] Venter C Agostoni C Arshad SH Ben-Abdallah M Du Toit G Fleischer DM Greenhawt M Glueck DH Groetch M Lunjani N Maslin K Maiorella A Meyer R Antonella M Netting MJ Ibeabughichi Nwaru B Palmer DJ Palumbo MP Roberts G Roduit C Smith P Untersmayr E Vanderlinden LA O’Mahony L. Dietary factors during pregnancy and atopic outcomes in childhood: a systematic review from the European Academy of Allergy and Clinical Immunology. Pediatr Allergy Immunol. 2020;31:889-912. 32524677 10.1111/pai.13303 PMC9588404

[63] Obata Y Furusawa Y Hase K. Epigenetic modifications of the immune system in health and disease. Immunol Cell Biol. 2015;93:226-232. 25666097 10.1038/icb.2014.114

[64] Han S-C Kang G-J Ko Y-J Kang H-K Moon S-W Ann Y-S Yoo E-S. Fermented fish oil suppresses T helper 1/2 cell response in a mouse model of atopic dermatitis via generation of CD4+CD25+Foxp3+ T cells. BMC Immunol. 2012;13:44. 22873180 10.1186/1471-2172-13-44 PMC3537649

[65] D’Vaz N Meldrum SJ Dunstan JA Lee-Pullen TF Metcalfe J Holt BJ Serralha M Tulic MK Mori TA Prescott SL. Fish oil supplementation in early infancy modulates developing infant immune responses. Clin Exp Allergy. 2012;42:1206-1216. 22805468 10.1111/j.1365-2222.2012.04031.x

[66] Gao Y Zhao C Wang W Jin R Li Q Ge Q Guan Y Zhang Y. Prostaglandins E2 signal mediated by receptor subtype EP2 promotes IgE production in vivo and contributes to asthma development. Sci Rep. 2016;6:20505. 26852804 10.1038/srep20505 PMC4744937

[67] Donato-Trancoso A Monte-Alto-Costa A Romana-Souza B. Olive oil-induced reduction of oxidative damage and inflammation promotes wound healing of pressure ulcers in mice. J Dermatol Sci. 2016;83:60-69. 27091748 10.1016/j.jdermsci.2016.03.012

[68] Romana-Souza B Monte-Alto-Costa A. Olive oil reduces chronic psychological stress-induced skin aging in mice through the NF-κB and NRF2 pathways. J Funct Foods. 2019;54:310-319.

[69] Cremonini E Wang Z Bettaieb A Adamo AM Daveri E Mills DA Kalanetra KM Haj FG Karakas S Oteiza PI. (-)-Epicatechin protects the intestinal barrier from high fat diet-induced permeabilization: implications for steatosis and insulin resistance. Redox Biol. 2018;14:588-599. 29154190 10.1016/j.redox.2017.11.002 PMC5691220

[70] Fritsche KL. The science of fatty acids and inflammation. Adv Nutr. 2015;6:293S-301S. 25979502 10.3945/an.114.006940 PMC4424767

[71] González F Considine RV Abdelhadi OA Acton AJ. Oxidative stress in response to saturated fat ingestion is linked to insulin resistance and hyperandrogenism in polycystic ovary syndrome. J Clin Endocrinol Metab. 2019;104:5360-5371. 31298704 10.1210/jc.2019-00987 PMC6773460

[72] Rocha DM Caldas AP Oliveira LL Bressan J Hermsdorff HH. Saturated fatty acids trigger TLR4-mediated inflammatory response. Atherosclerosis. 2016;244:211-215. 26687466 10.1016/j.atherosclerosis.2015.11.015

[73] Li J Wang Y Tang L de Villiers WJS Cohen D Woodward J Finkelman FD Eckhardt ERM. Dietary medium-chain triglycerides promote oral allergic sensitization and orally induced anaphylaxis to peanut protein in mice. J Allergy Clin Immunol. 2013;131:442-450. 23182172 10.1016/j.jaci.2012.10.011 PMC3563838

[74] Lopez-Garcia E Schulze MB Meigs JB Manson JE Rifai N Stampfer MJ Willett WC Hu FB. Consumption of trans fatty acids is related to plasma biomarkers of inflammation and endothelial dysfunction. J Nutr. 2005;135:562-566. 15735094 10.1093/jn/135.3.562

[75] Barcelos RCS Vey LT Segat HJ Benvegnú DM Trevizol F Roversi K Roversi K Dias VT Dolci GS Kuhn FT Piccolo J CristinaVeit J Emanuelli T Bürger ME. Influence of trans fat on skin damage in first-generation rats exposed to UV radiation. Photochem Photobiol. 2015;91:424-430. 25600099 10.1111/php.12414

[76] Ghanim H Abuaysheh S Sia CL Korzeniewski K Chaudhuri A Fernandez-Real JM Dandona P. Increase in plasma endotoxin concentrations and the expression of Toll-like receptors and suppressor of cytokine signaling-3 in mononuclear cells after a high-fat, high-carbohydrate meal: implications for insulin resistance. Diabetes Care. 2009;32:2281-2287. 19755625 10.2337/dc09-0979 PMC2782991

[77] Mahmud MR Akter S Tamanna SK Mazumder L Esti IZ Banerjee S Akter S Hasan MR Acharjee M Hossain MS Pirttilä AM. Impact of gut microbiome on skin health: gut-skin axis observed through the lenses of therapeutics and skin diseases. Gut Microbes. 2022;14:2096995. 35866234 10.1080/19490976.2022.2096995 PMC9311318

[78] Liu Y Liu Y Du Z Zhang L Chen J Shen Z Liu Q Qin J Lv H Wang H He L Liu J Huang Q Sun Y Otto M Li M. Skin microbiota analysis-inspired development of novel anti-infectives. Microbiome. 2020;8:85. 32503672 10.1186/s40168-020-00866-1 PMC7275423

[79] Baïz N Just J Chastang J Forhan A de Lauzon-Guillain B Magnier A-M Annesi-Maesano I; EDEN Mother-Child Cohort Study Group. Maternal diet before and during pregnancy and risk of asthma and allergic rhinitis in children. Allergy Asthma Clin Immunol. 2019;15:40. 31285746 10.1186/s13223-019-0353-2 PMC6589169

[80] Volynets V Louis S Pretz D Lang L Ostaff MJ Wehkamp J Bischoff SC. Intestinal barrier function and the gut microbiome are differentially affected in mice fed a western-style diet or drinking water supplemented with fructose. J Nutr. 2017;147:770-780. 28356436 10.3945/jn.116.242859

[81] Knight T Smith PK Soutter V Oswald E Venter C. Is the low pH of infant and toddler foods a concern? Pediatr Allergy Immunol. 2021;32:1103-1106. 33190325 10.1111/pai.13414

[82] Berni Canani R Carucci L Coppola S D’Auria E O’Mahony L Roth-Walter F Vassilopolou E Agostoni C Agache I Akdis C De Giovanni Di Santa Severina F Faketea G Greenhawt M Hoffman K Hufnagel K Meyer R Milani GP Nowak-Wegrzyn A Nwaru B Padua I Paparo L Diego P Reese I Roduit C Smith PK Santos A Untersmayr E Vlieg-Boerstra B Venter C. Ultra-processed foods, allergy outcomes and underlying mechanisms in children: an EAACI task force report. Pediatr Allergy Immunol. 2024;35:e14231. 39254357 10.1111/pai.14231

[83] Delaroque C Rytter H Bonazzi E Huillet M Ellero-Simatos S Chatonnat E Hao F Patterson A Chassaing B. Maternal emulsifier consumption alters the offspring early-life microbiota and goblet cell function leading to long-lasting diseases susceptibility. Nat Commun. 2025;16:6954. 40730751 10.1038/s41467-025-62397-3 PMC12307616

[84] David LA Maurice CF Carmody RN Gootenberg DB Button JE Wolfe BE Ling AV Devlin AS Varma Y Fischbach MA Biddinger SB Dutton RJ Turnbaugh PJ. Diet rapidly and reproducibly alters the human gut microbiome. Nature. 2014;505:559-563. 24336217 10.1038/nature12820 PMC3957428

[85] Ramne S Brunkwall L Ericson U Gray N Kuhnle GGC Nilsson PM Orho-Melander M Sonestedt E. Gut microbiota composition in relation to intake of added sugar, sugar-sweetened beverages, and artificially sweetened beverages in the Malmö Offspring Study. Eur J Nutr. 2021;60:2087-2097. 33030577 10.1007/s00394-020-02392-0 PMC8137620

[86] Uribarri J Woodruff S Goodman S Cai W Chen X Pyzik R Yong A Striker GE Vlassara H. Advanced glycation end products in foods and a practical guide to their reduction in the diet. J Am Diet Assoc. 2010;110:911-916. 20497781 10.1016/j.jada.2010.03.018 PMC3704564

[87] Liu L Zhang Z Xiao H Li Z Lin H. Dietary AGEs and food allergy: insights into the mechanisms of AGEs-induced food allergy and mitigation strategies. Crit Rev Food Sci Nutr. 2025;65:7853-7870. 40129068 10.1080/10408398.2025.2481990

[88] Clemente-Suárez VJ Beltrán-Velasco AI Redondo-Flórez L Martín-Rodríguez A Yáñez-Sepúlveda R Tornero-Aguilera JF. Global impacts of Western diet and its effects on metabolism and health: a narrative review. Nutrients. 2023;15:3106. doi: 10.3390/nu15122749. 37375654 10.3390/nu15122749 PMC10302286

[89] Toydemir G Gultekin Subasi B Hall RD Beekwilder J Boyacioglu D Capanoglu E. Effect of food processing on antioxidants, their bioavailability and potential relevance to human health. Food Chem X. 2022;14:100334. 35712535 10.1016/j.fochx.2022.100334 PMC9194584

[90] Ma X Nan F Liang H Shu P Fan X Song X Hou Y Zhang D. Excessive intake of sugar: an accomplice of inflammation. Front Immunol. 2022;13:988481. 36119103 10.3389/fimmu.2022.988481 PMC9471313

[91] Faggiani LD de França P Seabra SG Sabino EC Qi L Cardoso MA. Effect of ultra-processed food consumption on the gut microbiota in the first year of life: Findings from the MINA—Brazil birth cohort study. Clin Nutr. 2025;46:181-190. 39954456 10.1016/j.clnu.2025.01.030

[92] Sindi AS Stinson LF Lean SS Chooi Y-H Leghi GE Netting MJ Wlodek ME Muhlhausler BS Geddes DT Payne MS. Effect of a reduced fat and sugar maternal dietary intervention during lactation on the infant gut microbiome. Front Microbiol. 2022;13:900702. 36060782 10.3389/fmicb.2022.900702 PMC9428759

[93] Marrs T Jo J-H Perkin MR Rivett DW Witney AA Bruce KD Logan K Craven J Radulovic S Versteeg SA van Ree R McLean WHI Strachan DP Lack G Kong HH Flohr C. Gut microbiota development during infancy: impact of introducing allergenic foods. J Allergy Clin Immunol. 2021;147:613-621.e9. 33551026 10.1016/j.jaci.2020.09.042 PMC9169695

[94] McKenzie C Tan J Macia L Mackay CR. The nutrition-gut microbiome-physiology axis and allergic diseases. Immunol Rev. 2017;278:277-295. 28658542 10.1111/imr.12556

[95] Miyake Y Sasaki S Tanaka K Hirota Y. Consumption of vegetables, fruit, and antioxidants during pregnancy and wheeze and eczema in infants. Allergy. 2010;65:758-765. 20102358 10.1111/j.1398-9995.2009.02267.x

[96] Chatzi L Torrent M Romieu I Garcia-Esteban R Ferrer C Vioque J Kogevinas M Sunyer J. Mediterranean diet in pregnancy is protective for wheeze and atopy in childhood. Thorax. 2008;63:507-513. 18198206 10.1136/thx.2007.081745

[97] Ellwood P Asher MI Björkstén B Burr M Pearce N Robertson CF. Diet and asthma, allergic rhinoconjunctivitis and atopic eczema symptom prevalence: an ecological analysis of the International Study of Asthma and Allergies in Childhood (ISAAC) data. Eur Respir J. 2001;17:436-443. 11405522 10.1183/09031936.01.17304360

[98] Asnicar F Berry SE Valdes AM Nguyen LH Piccinno G Drew DA Leeming E Gibson R Le Roy C Khatib HA Francis L Mazidi M Mompeo O Valles-Colomer M Tett A Beghini F Dubois L Bazzani D Thomas AM Mirzayi C Khleborodova A Oh S Hine R Bonnett C Capdevila J Danzanvilliers S Giordano F Geistlinger L Waldron L Davies R Hadjigeorgiou G Wolf J Ordovás JM Gardner C Franks PW Chan AT Huttenhower C Spector TD Segata N. Microbiome connections with host metabolism and habitual diet from 1,098 deeply phenotyped individuals. Nat Med. 2021;27:321-332. 33432175 10.1038/s41591-020-01183-8 PMC8353542

[99] West CE Dunstan J McCarthy S Metcalfe J D’Vaz N Meldrum S Oddy WH Tulic MK Prescott SL. Associations between maternal antioxidant intakes in pregnancy and infant allergic outcomes. Nutrients. 2012;4:1747-1758. 23201845 10.3390/nu4111747 PMC3509518

[100] Yong TL Zaman R Rehman N Tan CK. Ceramides and skin health: new insights. Exp Dermatol. 2025;34:e70042. 39912256 10.1111/exd.70042

[101] Chapkin RS Ziboh VA Marcelo CL Voorhees JJ. Metabolism of essential fatty acids by human epidermal enzyme preparations: evidence of chain elongation. J Lipid Res. 1990;27:945-954. 3097227

[102] Choi EH. Skin barrier function in neonates and infants. Allergy Asthma Immunol Res. 2025;17:32-46. 39895601 10.4168/aair.2025.17.1.32 PMC11791375

[103] Conti A Rogers J Verdejo P Harding CR Rawlings AV. Seasonal influences on stratum corneum ceramide 1 fatty acids and the influence of topical essential fatty acids. Int J Cosmet Sci. 1996;18:1-12. 19245474 10.1111/j.1467-2494.1996.tb00131.x

[104] Walters RM Khanna P Chu M Mack MC. Developmental changes in skin barrier and structure during the first 5 years of life. Skin Pharmacol Physiol. 2016;29:111-118. 27161444 10.1159/000444805

[105] De Filippis F Paparo L Nocerino R Della Gatta G Carucci L Russo R Pasolli E Ercolini D Berni Canani R. Specific gut microbiome signatures and the associated pro-inflammatory functions are linked to pediatric allergy and acquisition of immune tolerance. Nat Commun. 2021;12:5958. 34645820 10.1038/s41467-021-26266-z PMC8514477

[106] Kishino S Takeuchi M Park S-B Hirata A Kitamura N Kunisawa J Kiyono H Iwamoto R Isobe Y Arita M Arai H Ueda K Shima J Takahashi S Yokozeki K Shimizu S Ogawa J. Polyunsaturated fatty acid saturation by gut lactic acid bacteria affecting host lipid composition. Proc Natl Acad Sci U S A. 2013;110:17808-17813. 24127592 10.1073/pnas.1312937110 PMC3816446

[107] Kapoor MP Yamaguchi H Ishida H Mizutani Y Timm D Abe A. The effects of prebiotic partially hydrolyzed guar gum on skin hydration: a randomized, open-label, parallel, controlled study in healthy humans. J Funct Foods. 2023;103:105494.

[108] Woodby B Penta K Pecorelli A Lila MA Valacchi G. Skin health from the inside out. Annu Rev Food Sci Technol. 2020;11:235-254. 31905017 10.1146/annurev-food-032519-051722

[109] Salem I Ramser A Isham N Ghannoum MA. The gut microbiome as a major regulator of the gut-skin axis. Front Microbiol. 2018;9:1459. 30042740 10.3389/fmicb.2018.01459 PMC6048199

[110] Trompette A Pernot J Perdijk O Alqahtani RAA Domingo JS Camacho-Muñoz D Wong NC Kendall AC Wiederkehr A Nicod LP Nicolaou A von Garnier C Ubags NDJ Marsland BJ. Gut-derived short-chain fatty acids modulate skin barrier integrity by promoting keratinocyte metabolism and differentiation. Mucosal Immunol. 2022;15:908-926. 35672452 10.1038/s41385-022-00524-9 PMC9385498

[111] Nguyen TD Hållenius FF Lin X Nyman M Prykhodko O. Monobutyrin and monovalerin affect brain short-chain fatty acid profiles and tight-junction protein expression in ApoE-knockout rats fed high-fat diets. Nutrients. 2020;12:1202. 32344633 10.3390/nu12041202 PMC7230324

[112] Li Y Dong J Xiao H Zhang S Wang B Cui M Fan S. Gut commensal derived-valeric acid protects against radiation injuries. Gut Microbes. 2020;11:789-806. 31931652 10.1080/19490976.2019.1709387 PMC7524389

[113] McCusker MM Grant-Kels JM. Healing fats of the skin: the structural and immunologic roles of the omega-6 and omega-3 fatty acids. Clin Dermatol. 2010;28:440-451. 20620762 10.1016/j.clindermatol.2010.03.020

[114] De Spirt S Stahl W Tronnier H Sies H Bejot M Maurette J-M Heinrich U. Intervention with flaxseed and borage oil supplements modulates skin condition in women. Br J Nutr. 2009;101:440-445. 18761778 10.1017/S0007114508020321

[115] Fujii M Ohyanagi C Kawaguchi N Matsuda H Miyamoto Y Ohya S Nabe T. Eicosapentaenoic acid ethyl ester ameliorates atopic dermatitis-like symptoms in special diet-fed hairless mice, partly by restoring covalently bound ceramides in the stratum corneum. Exp Dermatol. 2018;27:837-840. 29392772 10.1111/exd.13507

[116] Djuricic I Calder PC. Beneficial outcomes of Omega-6 and Omega-3 polyunsaturated fatty acids on human health: an update for 2021. Nutrients. 2021;13:2421. 34371930 10.3390/nu13072421 PMC8308533

[117] Jia T Qiao W Yao Q Wu W Kaku K. Treatment with docosahexaenoic acid improves epidermal keratinocyte differentiation and ameliorates inflammation in human keratinocytes and reconstructed human epidermis models. Molecules. 2019;24:3156. 31480216 10.3390/molecules24173156 PMC6749566

[118] Silva JR Burger B Kühl CMC Candreva T Dos Anjos MBP Rodrigues HG. Wound healing and omega-6 fatty acids: from inflammation to repair. Mediators Inflamm. 2018;2018:2503950. 29849484 10.1155/2018/2503950 PMC5925018

[119] Neukam K De Spirt S Stahl W Bejot M Maurette J-M Tronnier H Heinrich U. Supplementation of flaxseed oil diminishes skin sensitivity and improves skin barrier function and condition. Skin Pharmacol Physiol. 2011;24:67-74. 21088453 10.1159/000321442

[120] Losol P Rezwan FI Patil VK Venter C Ewart S Zhang H Arshad SH Karmaus W Holloway JW. Effect of gestational oily fish intake on the risk of allergy in children may be influenced by FADS1/2, ELOVL5 expression, and DNA methylation. Genes Nutr. 2019;14:20. 31244960 10.1186/s12263-019-0644-8 PMC6582528

[121] Moestrup KS Chen Y Schepeler T Schweiger PJ Jensen KB. Dietary control of skin lipid composition and microbiome. J Invest Dermatol. 2018;138:1225-1228. 29248545 10.1016/j.jid.2017.12.005

[122] Yamane T Kobayashi-Hattori K Oishi Y. A high-fat diet reduces ceramide synthesis by decreasing adiponectin levels and decreases lipid content by modulating HMG-CoA reductase and CPT-1 mRNA expression in the skin. Mol Nutr Food Res. 2011;55(Suppl 2):S186-S192. 21732532 10.1002/mnfr.201100144

